# Menstrual Pain and Elasticity of Uterine Cervix

**DOI:** 10.3390/jcm10051110

**Published:** 2021-03-07

**Authors:** Anjeza Xholli, Gianluca Simoncini, Sonja Vujosevic, Giulia Trombetta, Alessandra Chiodini, Mattia Francesco Ferraro, Angelo Cagnacci

**Affiliations:** 1 Academic Unit of Obstetrics and Gynecology, Maternal and Child Health (DiNOGMI), IRCCS Ospedale Policlinico San Martino, 16132 Genova, Italy; anj160583@yahoo.com (A.X.); alessandra1chiodini@gmail.com (A.C.); mattiafrancescoferraro@yahoo.it (M.F.F.); 2 Academic Unit of Obstetrics and Gynecology, Azienda Sanitaria Universitaria di Udine, 33100 Udine, Italy; dr.simoncini@gmail.com (G.S.); s.vujosevic@yahoo.com (S.V.); trombetta.giulia@hotmail.com (G.T.)

**Keywords:** chronic pelvic pain, dysmenorrhea, elastography, cervix, menstrual pain, tissue stiffness, elastography

## Abstract

Menstrual pain is consequent to intense uterine contraction aimed to expel menstrual flow through downstream uterine cervix. Herein it was evaluated whether characteristics of uterine cervix are associated with intensity of menstrual pain. Ultrasound elastography was used to analyze cervix elasticity of 75 consecutive outpatient women. Elasticity was related to intensity of menstrual pain defined by a Visual Analogue Scale (VAS). Four regions of interest (ROI) were considered: internal uterine orifice (IUO), anterior (ACC) and posterior cervical (PCC) compartment and middle cervical canal (MCC). Tissue elasticity, evaluated by color score (from 0.5 = blue/violet (low elasticity) to 3.0 = red (high elasticity), and percent tissue deformation was analyzed. Elasticity of IUO was lower (*p* = 0.0001) than that of MCC or ACC, and it was negatively related (R2 = 0.428; *p* = 0.0001) to menstrual VAS (CR −2.17; 95%CI −3.80, −0.54; *p* = 0.01). Presence of adenomyosis (CR 3.24; 95% CI 1.94, 4.54; *p* = 0.0001) and cervix tenderness at clinical examination (CR 2.74; 95% CI 1.29, 4.20; *p* = 0.0004), were also independently related to menstrual VAS. At post hoc analysis, women with vs. without menstrual pain had lower IUO elasticity, expressed as color score (0.72 ± 0.40 vs. 0.92 ± 0.42; *p* = 0.059), lower percent tissue deformation at IUO (0.09 ± 0.05 vs. 0.13 ± 0.08; *p* = 0.025), a higher prevalence of cervical tenderness at bimanual examination (36.2% vs. 9.5%; *p* = 0.022) and a higher prevalence of adenomyosis (46.5% vs. 19.9%; *p* = 0.04). These preliminary data indicate that IUO elasticity is associated with the presence and the intensity of menstrual pain. Mechanisms determining IUO elasticity are useful to be explored.

## 1. Introduction

Almost 85% of young women suffer from some degree of menstrual pain [1]. Pain intensity can be evaluated by a visual analogue scale (VAS) [2,3], and is called dysmenorrhea when it is intense, impacts on daily activities and requires medical treatment [1,4,5]. Even in its more severe forms, menstrual pain is very common and represents an important disturbance, capable of influencing a woman’s quality of life [1,6]. It is the consequence of intense myometrial contractions stimulated by endometrial prostaglandins [4]. Prolonged menstrual pain induces changes in brain activity resembling features of chronic pain [7,8] that may lead to persistence of disease and to insurgence of chronic pelvic pain [8,9,10]. Contractions increase intrauterine pressure aimed to expel menstrual blood through downstream uterine cervix [11]. A stiff cervix may obstacle menstrual flow more than an elastic one, possibly causing intense and painful contractions. Ultrasound elastography was used to evaluate tissue stiffness/elasticity of breast and muscle [12], thyroid [13], liver [14], prostate [14], pancreas [15], uterine myomas and adenomyosis [16,17,18]. Application of elastography to uterine cervix has been mainly confined to obstetrics [19,20,21,22], stiffness modifications being used to define the risk of preterm birth [23,24] or to set the time for labor induction [25]. Rarely, elastography was used to investigate uterine cervix outside pregnancy [26,27,28,29,30,31,32]. In the present study, we evaluated whether elasticity of the uterine cervix is related to the intensity of menstrual pain.

## 2. Experimental Section

An observational study was performed between October 2017 and July 2018 in women of an outpatient service for contraception at a University Hospital. The study did not involve any intervention outside common clinical practice, but each woman signed a written informed consent for the anonymous use of her data in clinical research. The Institutional Review Board IRB gave consent to direct data publication. Cycling women, 18 to 45 years of age were included. Women who were pregnant, with any type of oncological disease, with an intrauterine device, or on hormonal contraception were excluded. Among 99 screened women, 20 women were on hormonal contraceptives and were excluded. The remaining 79 were considered.

**Figure 1:**
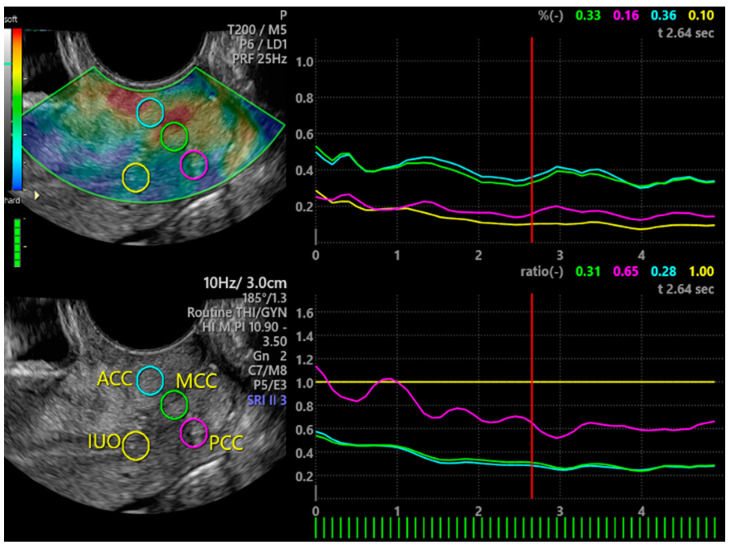
Elastography of uterine cervix. On the left, the two vertical bars indicate the colorimetric scale (upper bar) and the control bar (lower bar) that when full green indicates optimal compression force. Circles indicate ROIs. On the right are the numerical index and graphical representation of ROI’s percent tissue deformation (upper panel) and numerical index and graphical representation of ratio between IUO percent tissue elasticity (as reference) and other ROIs. ACC = anterior cervical compartment; PCC = posterior cervical compartment; IUO = internal uterine orifice; MCC = middle cervical canal.

Demographic and clinical data were collected for each woman. Presence of heavy menstrual bleeding identified as menstrual blood loss >80 mL, was later confirmed by the pictorial method [33]. A 10 cm VAS was used to measure intensity of menstrual pain, perceived by women in the last 3 menstrual cycles with the use of no medication [1,6]. Each woman underwent vaginal bimanual examination, to evaluate cervical stiffness and tenderness at passive mobilization. Presence of gynecological diseases such as uterine myomas, adenomyosis and endometriosis were evaluated by patient history, bimanual examination and ultrasonography. Ultrasonographic criteria for the diagnosis of ovarian and pelvic endometriosis [34,35,36], uterine myomas and adenomyosis [37] were used. Ultrasound investigations were performed by an expert trained practitioner (A.X.), who was blind about the woman menstrual pain, using a Voluson E10 General Electric (GE, General Electric Company, Boston, Massachusetts, USA) instrument with a transvaginal probe (TSV) GE RIC 59D) and a proper software for elastography (Voluson E10 BT16, General Elcetric Company, Boston Massachussetts, USA). For each woman, longitudinal (L), transverse (T) and antero-posterior (AP) diameter of the uterus, length and transverse diameter of the cervix, and elasticity of different cervical compartments was obtained. Uterus volume (cm^3^) was calculated by the ellipsoid formula (L × T × AP × 0.5223). Tissue elasticity was obtained by strain elastography (SE)(Figure 1). SE is based on differences in elasticity of various tissues in both physiological and pathological conditions [31,32]. It measures tissue deformation or displacement generated by an applied pressure. During image acquisition, vaginal probe was positioned in the anterior vaginal fornix and a B-Mode sagittal view of the cervix was obtained and displayed alongside to facilitate images interpretation [30]. The operator performed a series of about 5 compression and decompression cycles, using sub-centimetric excursions perpendicular to the axis of the cervical canal [19,25]. A control bar of the ultrasound processing program indicated, in real time, optimal compression force (Figure 1). Regions of interest (ROIs) with a circular area of 19.6 mm^2^ were placed in the middle of the anterior cervical compartment (ACC), in the middle of the posterior cervical compartment (PCC), in the middle portion of the cervical canal (MCC) and at the internal uterine orifice (IUO) (Figure 1). SE evaluations were recorded on clips and analyzed afterwards. Results were calculated at optimal compression force. Tissue elasticity coded with a scale ranging from violet/blue (low) to red (high), with yellow/green as intermediate elasticity, was evaluated by three independent scorers, who were blind about menstrual VAS value. A predefined value was assigned on the basis of the colorimetric scale on the whole spectrum (from the value 0.5 = blue/violet to the value 3.0 = red) [31,32]. Mean value of the 3 scorers was used. The SE software, (General Electrics Company, Boston, Massachussets, USA in use also provided a numerical index of the ROI’s percent tissue deformation (Figure 1). This value was also considered in statistical analysis. Ratios of both color score elasticity and percent tissue deformation of different ROIs were calculated and compared.

Analysis of variance (ANOVA) for repeated measures with subjects as replicates was used to compare elasticity of the different cervix compartments. Linear regression analysis was used to test the relation of menstrual pain VAS value (dependent variable) and factors (independent) expressed by continuous or categorical data. Continuous data were age (years), age at menarche (years), uterine volume (cm^3^), cervix length and diameter (cm), color score of elasticity and percent deformation of the four cervical ROIs. Ratios of color score elasticity or percent deformation of different ROIs were also considered. Categorical data entered as dummy variables, were previous pregnancy vs. no, previous caesarean section vs. no, cervical stiffness at bi-manual investigation vs. no, tenderness at cervix mobilization during bi-manual investigation vs. no, presence of heavy menstrual periods vs. no, presence of endometriosis, myoma, or adenomyosis vs. no. Variables, that at simple regression analysis were significantly related to menstrual pain, were entered in a multiple regression model in order to define those factors that were independently related to intensity of menstrual pain. At a post hoc analysis, 59 women were suffering and 21 were not suffering from menstrual pain (VAS = 0). Values of women with any intensity of menstrual pain were compared to those of women without menstrual pain. Means of the two groups were compared by the Student’s *t*-test, while frequencies were compared by contingency tables and the Chi-squared test.

Many studies investigating cervix elasticity included from 20 to 74 women [20,23,30,31,32]. We estimated that a similar number of women was sufficient to define elasticity of the cervix and its relation to the intensity of menstrual pain. Statistical analysis was performed with the StatView program (SAS Institute Inc. Cary, NC, USA). Data are expressed as mean ± standard deviation (SD). A *p*-value < 0.05 was considered as significant.

## 3. Results

### 3.1. Study Participants

Data of study participants are reported in Table 1. Mean VAS value of menstrual pain was 4.55 ± 3.7 in the whole sample of women, but it was 6.19 ± 2.9 in women with pain and by definition it was 0 in women without menstrual pain. Among women with menstrual pain, 13 women had a VAS < 4, 18 had a VAS between 4 and 7, and 27 had a VAS ≥ 7. There was no difference between women with and without menstrual pain, but in the former a higher rate of adenomyosis, and cervix tenderness at bimanual examination was obtained (*p* = 0.022) (Table 1).

### 3.2. Elastography of the Cervix

Compartments of the cervix showed a different elasticity (Table 2). IUO had a lower elasticity (*p* = 0.0001) than MCC and ACC.

At linear regression analysis, intensity of menstrual pain was negatively related to color score elasticity of IUO, PCC, and elasticity ratio of IUO/MCC, IUO/ACC, and PCC/ACC (Table 3). Menstrual pain was also negatively related to percent tissue deformation of IUO and to the percent tissue deformation ratio IUO/ACC (Table 3). In addition, intensity of menstrual pain was positively related to the presence of adenomyosis, pelvic endometriosis, heavy menstrual bleeding, and cervical tenderness at clinical examination (Table 3).

When these single factors were entered into multiple regression analysis, only IUO elasticity at color score (CR −2.17; 95% CI −3.80, −0.54; *p* = 0.01), cervix tenderness (CR 2.74; 95% CI 1.29, 4.20; *p* = 0.0004), and presence of adenomyosis (CR 3.24; 95% CI 1.94, 4.54; *p* = 0.0001) remained significantly related to the intensity of menstrual pain (R2 0.428; *p* = 0.0001) (Table 3).

### 3.3. Post Hoc Groups Comparison

Heterogeneity of cervix elasticity was different in women with (*n* = 58) and without (*n* = 21) menstrual pain. In women with menstrual pain, IUO had a lower elasticity than both MCC and ACC (*p* = 0.0001), while in women with no menstrual pain, IUO had a lower elasticity of MCC only (*p* = 0.046).

Comparisons between groups showed that IUO color score elasticity (*p* = 0.059) and percent tissue deformation (*p* = 0.025) was lower in women with than without menstrual pain (Table 2). Ratio IUO/MCC of color score elasticity (*p* = 0.021) or percent tissue deformation (*p* = 0.05) was also lower in women with than without menstrual pain (Table 2). Similarly, ratio IUO/ACC of color score elasticity (*p* = 0.015) or percent tissue deformation (*p* = 0.035) was lower in women with than without menstrual pain (Table 2).

## 4. Discussion

The present study indicates that intensity of menstrual pain is related to elasticity of IUO, higher pain being present with lower IUO elasticity. Additional related factors to intensity of menstrual pain are the presence of adenomyosis and a tender cervix, at bimanual examination.

SE allows evaluation of tissue elasticity by measuring ROIs tissue deformation during compressive and decompressive forces [31,32]. Elasticity of uterine cervix was seldom investigated in gynecological conditions, and it emerged that elasticity of various cervical area or ROIs is different [27,29,30,31,38]. In our study, ROIs of uterine cervix showed a different tissue elasticity, the inner part of tissue around the IUO showing a lower elasticity than other areas, particularly the MCC and ACC. Besides possible confounding related to the method of investigation, it is likely that difference in elasticity is the consequence of a different anatomical conformation of the cervix at the IUO. The presence of radial collagen fibers, along with a robust bundle of circular collagen fibers, is peculiar of this anatomical area [39]. In addition, 50% of this area tissue is composed by circular muscular cells responsive to oxytocin and neurotransmitters [40]. These peculiarities make the IUO mechanically apt to containment. It represents an area that, more than others, counteracts dilatative forces exerted by the fetus, during pregnancy [39,40,41] and likely, by menstrual blood, during menses. Lower elasticity at the IUO indicates a structure harder to deform by an external force, but also by an internal dilatative force, as previously reported in pregnancy [21,41]. The present study shows that a lower elasticity at the IUO is present in women suffering from menstrual pain, with an inverse linear relation between the degree of elasticity and the intensity of pain. This relation was not observed for other ROIs, including the MCC. IUO/MCC, or even IUO/ACC elasticity ratios, were lower in women with than without menstrual pain. These differences indicate that heterogeneity of tissue elasticity within the cervix is magnified in women with menstrual pain.

Elasticity of IUO was independently related to intensity of menstrual pain even when other risk factors, such as heavy menstrual bleeding, endometriosis or adenomyosis, were considered. Interestingly, when elasticity of IUO was taken into account, most risk factors lost their relations with menstrual pain. Only adenomyosis remained independently related to pain intensity.

There are several weaknesses in this study. Elastography was applied to different ROIs and multiple comparisons were made. Accordingly, some of them can be significant only by chance. SE does not allow absolute quantification of elasticity, that can only be appropriately evaluated on cervix tissue specimens in vitro. SE results are conditioned by the force the operator applies during the evaluation [31,32]. Analysis was optimized by performing the evaluation at optimal compression, as indicated in real time by the software in use. Data obtained at IUO were confirmed by performing ratios between IUO and other ROIs. Assuming that the applied external force is equivalent across the cervix, ratio values are independent on the applied external force [42]. In addition, it has been reported that evaluation of IUO’s elasticity is less variable than that of other areas of the cervix [19]. The results are rather consistent and in agreement with published studies showing that this area has an anatomical composition different from other regions of the cervix [39,40]. Independent readings of the 3 examiners were used as a mean value and not separately. Accordingly, we did not evaluate interrater reliability. This is going to be evaluated in future studies.

## 5. Conclusions

Overall, the data indicate that menstrual pain is associated with a lower elasticity of the cervical tissue around the IUO. Stability over time of these preliminary data should be tested and confirmed in additional studies. Whether confirmed, mechanisms determining IUO elasticity may be useful to be explored.

## Figures and Tables

**Table 1:** Mean (± SD) values of enrolled women, also divided at post hoc analysis in women with and without menstrual pain. Significance of comparison between the two groups is reported.

	Total (*n* = 79)	Pain (*n* = 58)	No Pain (*n* = 21)	*p*-Value
Menstrual pain VAS	4.55 ± 3.7	6.19 ± 2.9	0	0.0001
Heavy Menstrual Bleedings (%)	16.4	22.4	14.2	0.425
Age (years)	34.9 ± 8.7	34.8 ± 9.2	35.4 ± 7.2	0.791
Menarche (years)	12.4 ± 1.4	12.4 ± 1.4	12.5 ± 1.5	0.908
Nulliparous (%)	51.9	51.7	52.4	0.935
Caesarean Section (%)	12.6	13.8	9.5	0.614
Adenomyosis (%)	39.2	46.5	19.0	0.04
Myomas (%)	22.8	15.5	42.8	0.058
Ovarian endometriosis (%)	13.9	13.8	14.3	0.955
Pelvic endometriosis (%)	16.5	18.9	4.8	0.125
Uterine volume (cm^3^)	87.7 ± 77.5	92.8 ± 88.7	73.3 ± 26.9	0.328
Cervix length (mm)	28.5 ± 4.1	28.7 ± 4.5	28.1 ± 3.5	0.657
Cervix Transverse diameter (mm)	25.5 ± 4.3	26.1 ± 4.3	25.0 ± 4.4	0.481
Clinical cervical stiffness (%)	43.0%	48.3%	28.5%	0.118
Tenderness at cervix mobilization (%)	29.1%	36.2%	9.5%	0.022

VAS = visual analogue scale.

**Table 2:** Mean (± SD) color score elasticity, percent tissue deformation and ratios of different regions of interest of the cervix: Data of women with and without menstrual pain are also reported along with their comparison.

Cervical Compartments	Total (*n* = 79)	Pain (*n* = 58)	No Pain (*n* = 21)	*p*-Value
ACC Elasticity	1.21 ± 0.27	1.22 ± 0.26	1.17 ± 0.30	0.779
ACC % Deformation	0.17 ± 0.09	0.18 ± 0.10	0.15 ± 0.08	0.285
PCC Elasticity	0.59 ± 0.38 *	0.58 ± 0.38 *	0.65 ± 0.38 ^†^	0.465
PCC % Deformation	0.11 ± 0.07 *	0.10 ± 0.07 *	0.11 ± 0.06	0.791
IUO Elasticity	0.77 ± 0.41 *	0.72 ± 0.40 *	0.92 ± 0.42	0.059
IUO % Deformation	0.10 ± 0.06 *	0.09 ± 0.05 *	0.13 ± 0.08	0.025
MCC Elasticity	1.10 ± 0.35	1.12 ± 0.29	1.07 ± 0.48	0.612
MCC % Deformation	0.17 ± 0.09	0.17 ± 0.09	0.19 ± 0.11 ^‡^	0.386
IUO/MCC Elasticity	0.83 ± 0.70	0.72 ± 0.64	1.13 ± 0.98	0.021
IUO/MCC % Deformation	0.68 ± 0.43	0.63 ± 0.31	0.84 ± 0.62	0.050
IUO/ACC Elasticity	0.71 ± 0.77	0.59 ± 0.32	1.06 ± 1.3	0.015
IUO/ACC % Deformation	0.77 ± 0.76	0.66 ± 0.68	1.06 ± 0.88	0.035
IUO/PCC Elasticity	1.78 ± 1.54	1.62 ± 1.27	2.18 ± 2.01	0.160
IUO/PCC % Deformation	1.42 ± 1.72	1.37 ± 1.89	1.56 ± 1.05	0.666
PCC/ACC Elasticity	0.50 ± 0.32	0.47 ± 0.32	0.57 ± 0.29	0.810
PCC/ACC % Deformation	0.13 ± 0.24	0.101 ± 0.07	0.198 ± 0.46	0.122

ACC = anterior cervical compartment; PCC = posterior cervical comportment; MCC = middle cervical canal; IUO = internal uterine orifice. * *p* = 0.0001 vs. corresponding ACC and MCC compartments; ^†^ *p* = 0.005 vs. corresponding ACC and MCCl; ^‡^ *p* = 0.005 vs. corresponding PCC and *p* = 0.046 vs. corresponding IUO.

**Table 3:** Results of single and multiple linear regression analyses (R2 = 0.428; *p* = 0.0001) performed between intensity of menstrual pain expressed as visual analogue scale (VAS) value and related factors, among which include tissue color score elasticity and tissue percent deformation.

	Single Regression			Multiple Regression		
Factor	CR	95% CI	R2; *p*-Value	CR	95% CI	*p*-Value
Adenomyosis (y/*n*)	3.63	2.12; 5.14	0.230; 0.0001	3.24	1.94; 4.54	0.0001
Pelvic endometriosis (y/*n*)	4.08	2.02; 6.14	0.168; 0.0002	/	/	NS
Heavy Menstrual Bleeding (y/*n*)	4.13	2.07; 6.19	0.171; 0.0001	/	/	NS
Cervix Tenderness (y/*n*)	3.55	1.88; 5.21	0.190; 0.0001	2.74	1.29; 4.20	0.0004
IUO Elasticity	−2.92	−4.73; −1.19	0.100; 0.002	−2.17	−3.80; −0.54	0.01
PCC Elasticity	−2.86	−4.40; −0.11	0.054; 0.04	/	/	NS
IUO % Deformation	−17.74	−30.8; −4.63	0.086; 0.009	/	/	NS
IUO/MCC Elasticity	−1.69	−2.82; −0.56	0.092; 0.004	/	/	NS
IUO/ACC Elasticity	−1.383	−2.43; −0.33	0.069; 0.011	/	/	NS
PCC/ACC Elasticity	−2.932	−5.48; −0.38	0.064; 0.026	/	/	NS
IUO/ACC % Deformation	−1.198	−2.28; −0.12	0.059; 0.030	/	/	

CR = coefficient of regression; CI = confidence Interval; ACC = anterior cervical compartment; PCC = Posterior cervical compartment; IUO = internal uterine orifice; MCC = middle cervical canal. y/n = yes/no; NS = not significant

## Data Availability

The datasets used and/or analysed during the current study are available from the corresponding author on reasonable request.
